# High Frequency of Tumor Propagating Cells in Fusion-Positive Rhabdomyosarcoma

**DOI:** 10.3390/genes12091373

**Published:** 2021-08-31

**Authors:** Melanie Generali, Sampoorna Satheesha, Peter K. Bode, Debora Wanner, Beat W. Schäfer, Elisa A. Casanova

**Affiliations:** 1 Center for Therapy Development and Good Manufacturing Practice, Institute for Regenerative Medicine (IREM), University of Zurich, 8044 Zurich, Switzerland; melanie.generali@irem.uzh.ch (M.G.); debora.wanner@irem.uzh.ch (D.W.); 2 Department of Oncology and Children’s Research Center, University Children’s Hospital Zurich, 8032 Zurich, Switzerland; sampoorna.satheesha@gmail.com; 3 Department of Pathology and Molecular Pathology, University Hospital Zurich, University of Zurich, 8091 Zurich, Switzerland; PeterKarl.Bode@usz.ch; 4 Division of Trauma Surgery, Center for Clinical Research, University Hospital Zurich, University of Zurich, 8091 Zurich, Switzerland

**Keywords:** Rhabdomyosarcoma, PAX3-FOXO1, tumor propagating stem-like cells, cancer stem cells, tumor heterogeneity

## Abstract

Rhabdomyosarcoma (RMS) is the most common soft tissue sarcoma in children. Fusion-positive RMS (FPRMS), expressing the PAX3/7-FOXO1, has a worse prognosis compared to the more common fusion-negative RMS (FNRMS). Although several studies reported hierarchical organization for FNRMS with the identification of cancer stem cells, the cellular organization of FPRMS is not yet clear. In this study we investigated the expression of key stem cell markers, developed a sphere assay, and investigated the seven most common FPRMS cell lines for subpopulations of tumor propagating cancer stem-like cells, also called cancer stem cells (CSCs). Moreover, loss- and gain-of-functions of the stem cell genes SOX2, OCT4, and NANOG were investigated in the same cells. Single-cell clonal analysis was performed in vitro as well as in vivo. We found that no stable CSC subpopulation could be enriched in FPRMS. Unlike depletion of PAX3-FOXO1, neither overexpression nor siRNA-mediated downregulation of SOX2, OCT4, and NANOG affected physiology of RMS cells. Every single subclone-derived cell clone initiated tumor growth in mice, despite displaying considerable heterogeneity in gene expression. FPRMS appears to contain a high frequency of tumor propagating stem-like cells, which could explain their higher propensity for metastasis and relapse. Their dependency on PAX3-FOXO1 activity reinforces the importance of the fusion protein as the key therapeutic target.

## 1. Introduction

To understand tumor origin, maintenance, progression and heterogeneity, two models have been postulated in the past: the stochastic or clonal evolution model and the hierarchical or cancer stem cell model [1]. For the development of more effective cancer therapies, it is of fundamental importance to understand the extent of heterogeneity and associated hierarchy within a tumor.

Cancer stem cells (CSCs) possess the ability for unlimited self-renewal and to generate the heterogeneous and differentiated cell lineages that form the tumor bulk [2]. Because of these unique characteristics, CSCs are also called tumor initiating cells or tumor propagating cells. The frequency of CSCs within the tumor depends on the tumor type [3] and tends to increase with malignant progression [4,5,6,7]. Based on phenotypic traits such as cell surface markers and on functional properties such as sphere formation, quiescence, and drug resistance, CSCs can nowadays be isolated from a number of primary tumors or cell lines and can be maintained and enriched in vitro, facilitating their molecular characterization.

Rhabdomyosarcoma (RMS) is the most frequent soft tissue sarcoma in children (reviewed in [8]). RMS shares features with skeletal muscle. Based on genetic analysis, it can be divided into two subgroups: fusion-negative or embryonal RMS (FNRMS) and fusion-positive or alveolar RMS (FPRMS). FNRMS has higher incidence, is less aggressive, and mainly affects children aged 0–5 years [9]. On the other hand, FPRMS occurs more frequently in adolescents and young adults and is more aggressive, with a high risk of relapse and generally worse prognosis [10,11,12]. The oncogenic driver of FPRMS is the result of specific chromosomal translocations, that generate a chimeric transcription factor PAX3-FOXO1 or PAX7-FOXO1 [13,14]. The fusion protein is the main recurrent genetic alteration [15,16], which has a fundamental role in FPRMS cell maintenance [17,18,19,20,21]. It actively contributes to the undifferentiated myogenic phenotype typical of RMS cells by suppressing the transcriptional activity of *MyoD*-target genes [22]. Additionally, epigenetic modulation by PAX3-FOXO1 target genes, JARID2 and EZH2, might contribute to inhibition of myogenic differentiation in RMS cells [23]. Similar features characterize transcription factors that regulate stemness in embryonic stem cells and induce reprogramming of differentiated cells into pluripotent stem cells (iPSC). The core stem cell factors OCT4 and SOX2, together with KLF4 and c-MYC, are sufficient for this phenomenon [24,25,26]. Not surprisingly, most of these reprogramming factors are upregulated in cancer (stem) cells, where they maintain the tumorigenic state and sustain cellular self-renewal [27,28,29]. Upregulation of SOX2 and OCT4 has also been observed in the population of FNRMS which was identified by functional sphere formation assays [30].

Although CSCs have already been identified in some sarcomas [31,32,33], and in FNRMS in particular [30], it is not known whether the aggressive FPRMS subtype possesses a subpopulation of CSCs. Therefore, in this study, we aim to use various in vitro and in vivo assays to assess whether FPRMS cell lines contain CSCs and thereby provide us with a better understanding of its biology.

In this study, no exclusive subpopulation of cells with stem-like cell potential could be defined in the seven common FPRMS cell lines. On the contrary, we present evidence that in FPRMS each cell possesses self-renewing and tumor initiating properties. Our data underline the notion that survival and maintenance of FPRMS cells is strictly PAX3-FOXO1 dependent. Hence, the fusion protein defines a high frequency of tumor initiating stem-like cells in FPRMS, which could explain the higher metastatic and relapse potential of this subgroup.

## 2. Materials and Methods

### 2.1. Cell Lines

FPRMS cell lines, RH4, RH41 (both kindly provided by Peter Houghton, Greehey CCRI, San Antonio, TX, USA), RH3, RH5 (kindly provided by Susan Ragsdale, St. Jude Children’s Research Hospital, Memphis, TN, USA), CW9019 (kindly provided by Soledad Gallego, Hospital Universitari Vall d’Hebron, Barcelona, Spain), RMS13 (kindly provided by Roland Kappler, Ludwig-Maximilians-Universität München, Munich, Germany), and RH30 (purchased from the American Type Cell Culture Collection, LGC Promochem, Molsheim Cedex, France) were routinely maintained in DMEM supplemented with 10% fetal bovine serum (FBS), 2 mM L-glutamine and 100 U/mL penicillin/streptomycin (referred to as a complete medium).

### 2.2. Sphere Assay, Transgenic FPRMS Cell Lines, and siRNA

Cells were seeded at clonal dilution (1 cell/μL) into Petri dishes and cultured in sphere medium consisting of DMEM/HAM’s F12, 20% knockout Serum Replacement (KSR) (GIBCO), 2 mM L-glutamine, 100 U/mL penicillin/streptomycin (P/S) either supplemented with 10 ng/mL bFGF or with 10 ng/mL bFGF, EGF, and PDGF (R&D Systems) (ALL media). At each passage, spheres were quantified and passaged at clonal density.

Cell lines stably overexpressing NANOG, OCT4, and SOX2 were generated using jetPRIME Transfection Reagent (Polyplus) and the following plasmids: control EGFP pEGIP (Addgene no. 26777 [34]), pSin-EF-Sox2-Pur, pSin-EF-Nanog-Pur, pSin-EF-Oct4-Pur (all from Addgene no. 16577–16579 [26]). The plasmids used for the overexpression of the stem cell genes contained a puromycin-resistance gene. Puromycin (1 µg/mL) selection was initiated 48 h after transfection and fresh complete medium with antibiotic was replaced during the first 3 days. Stable, transgenic FPRMS cell lines were expanded, analyzed for their overexpression and used for further experiments. Overexpression was demonstrated at RNA and protein levels.

For knockdown, cells were transfected using INTERFERin siRNA Transfection Reagent (Polyplus). Silencer^®^ Select siRNAs (Ambion, Life technologies, Carlsbad, CA, USA) against SOX2 (iSOX2: s13294), PAX3-FOXO1 (siP2F [35]), and scrambled control (Silencer Negative Control No. 2 siRNA) were used at a final concentration of 5nM. Cells were harvested after 48 h and 72 h for functional assays. Gene silencing was controlled at RNA and protein levels. The pluripotent embryonal carcinoma NTERA-2 cell line was used for testing the siRNAs-mediated silencing of the core stem cell genes.

### 2.3. Cancer Stem Cell Markers and Separation of ALDH-Positive Cells

For CSC marker analysis, single-cell suspensions were incubated at room temperature for 30 min with a 1:10 diluted fluorochrome-labeled antibody for: CD133/2-APC, CD133/1-APC (both Miltenyi), CD44-FITC, CXCR4-APC (both BD Bioscience). Unstained cells were used for setting the gates and specificity of the antibodies was confirmed with positive and negative cell lines. Flow cytometric analysis was performed on a BD FACS Canto II instrument (BD Biosciences). Data were collected with DIVA software (BD Biosciences) and analyzed with FlowJo software (TreeStar Inc., Ashland, OR, USA).

To monitor ALDH activity, the Aldefluor assay system (Stem Cell Technologies) was used according to manufacturer’s instructions. Briefly, 10^6^ cells/mL were incubated with activated ALDH substrate for 40 min at 37 °C. Using identical conditions as a negative control for each experiment, a set of cells was stained with ALDH inhibitor DEAB. For all experiments the DEAB-treated population was used to set the gates, as described in the Aldefluor^®^ assay manufacturer’s instructions. ALDH-positive cells were detected with FACS, as described previously. Aldefluor^®^-labeled FPRMS cells were sorted with FACS Aria III and fractioned into ALDH-positive and -negative. Control cells were unstained cells processed through the FACS machine and collected without sorting for specific subpopulations. The three populations were cultured either as monolayer and examined for ALDH activity after 3, 5, 6, and 10 days or as spheres, as described above.

### 2.4. Proliferation, Viability, Clonogenicity, PKH26 Staining, and Cell Cycle Analysis

For proliferation assay, Cell Proliferation ELISA, BrdU assay (Roche Applied Science, Penzberg, Germany) was used following the manufacturer’s instruction. For knockdown experiments the data were normalized with the scrambled control. For cell viability assay, cell proliferation reagent WST-1 (Roche Applied Science) was used following the manufacturer’s instruction. All experiments were performed in triplicates. For clonogenicity assay, 2000 cells were seeded on 6-well plates in triplicates. Cells were cultured with complete medium and allowed to grow until clones were visible. Cells were stained with crystal violet and clones were quantified. For asymmetrical division analysis, cells were stained with PKH26 Red Fluorescent Cell Linker Kit (Sigma-Aldrich, St. Louis, MO, USA) following the manufacturer’s instructions. At day 0, unstained and PKH26-stained cells were assessed with flow cytometry after which PKH26 staining was visualized at day 1, 3, 7, and 14. For cell cycle analysis, siRNA-treated cells were harvested after 24, 48, and 72 h and fixed in ice-cold 70% ethanol. Cells were resuspended in propidium iodide solution (PBS, 1% Triton X-100, 100 mg/mL RNaseA, 1 mg/mL PI), and processed for FACS analysis as previously described.

### 2.5. Real-Time PCR

Total RNA was extracted using RNeasy Mini Kit (Qiagen) and reverse transcription was carried out with the high-capacity cDNA reverse transcription kit (Life Technologies) according to the manufacturer’s instructions. Quantitative Real-Time PCR (qRT-PCR) was performed using commercially available mastermix and TaqMan Gene Expression Assays (Life Technologies, Supplemental Table S1). Reactions were run using standard conditions on an ABI 7900 HT Real-Time PCR machine and the data were analyzed with SDS 2.2 software. CT values were normalized to GAPDH or HMBS housekeeping genes. Relative expression levels of the genes were calculated using the ΔΔCt method [36]. All experiments were performed in triplicate and repeated at least three times.

### 2.6. Western Blotting, Immunofluorescence, and Immunohistochemical Analysis

For Western blot, cells were lysed with RIPA buffer (50 mM Tris-Cl, pH 6.8, 100 mM NaCl, 1% Triton X-100, 0.1% SDS) and supplemented with a Complete Mini Protease Inhibitor Cocktail (Roche Applied Sciences). Protein concentration was measured with a Pierce^®^ BCA protein Assay Kit (Thermo Scientific, Waltham, MA, USA). Proteins were identified by SDS-PAGE and Western blotting using antibodies reported in Supplemental Table S2. Signal was detected by chemiluminescence using SuperSignal West Femto Maximum Sensitivity Substrate (Thermo Scientific). Stem cell antibodies (NANOG, OCT4, SOX2) were titrated using the pluripotent embryonal carcinoma cell line NTERA2, which highly express all pluripotency genes. For immunofluorescence analysis, FPRMS cells were grown on chamber slides, fixed with 4% paraformaldehyde (Carl Roth, Arlesheim), and incubated over night at 4 °C with primary antibodies (Supplemental Table S2). Stem cell antibodies (NANOG, OCT4, SOX2) were titrated for immunofluorescence staining using the pluripotent embryonal carcinoma cell line NTERA2, which highly expresses all pluripotency genes. FPRMS staining was analyzed with an Axioskop2 mot plus fluorescence microscope (Zeiss, Jena, Germany). For immunohistochemical analysis of xenografts, three-micron thick sections of blocks of formalin-fixed, paraffin-embedded tissue were mounted on glass slides (SuperFrost Plus), deparaffinized, rehydrated and stained with hematoxylin and eosin (H&E) using standard histological techniques. Immunohistochemical staining was performed with the Ventana Benchmark automated staining system (Ventana Medical Systems) using Ventana UltraView DAB reagents; antibodies are listed in Supplemental Table S2. All primary antibodies were diluted in Ventana diluent. Slides were counterstained with hematoxylin, dehydrated and mounted.

### 2.7. Generation of Tumor Xenografts

NODscid/Hsd2 mice (Jackson Laboratories) were used in this present study. The animal housing and experimental protocols were approved by the Cantonal Veterinary Office, Zurich, Switzerland, under license No. ZH 208/2012 and were in accordance with Swiss Animal Protection Laws and conform to European Directive 2010/63/EU of the European Parliament and of the Council on the Protection of Animals used for Scientific Purposes. In vivo limiting dilution with RH30 cells: 10^5^ (*n* = 3), 10^4^ (*n* = 4), and 100 cells (*n* = 5) were injected intramuscularly (i.m.) into the left leg of NOD/Scid mice. Tumor growth was monitored over a period of 180 days. Tumor size was determined every 4 days by measuring two diameters (d_1_ and d_2_) in right angles of both legs with a caliper and volumes were calculated using the formula: V = [4/3 π 1/2(d_1_ + d_2_)]_left leg_ − [4/3 π 1/2 (d_1_ + d_2_)]_right leg_

For secondary tumors, primary xenografts were isolated and dissociated as described in [37] for 30 min at 37 °C in a rotator oven. An aliquot of each cell suspension was analyzed by flow cytometry for determining viable (7-AAD negative), EGFP positive cells. Based on these data dilutions with 10^4^ cells were prepared and injected i.m. into NODscid/Hsd2 mice.

### 2.8. Statistical Analysis

Statistical analysis was conducted using Student’s *t*-test with the GraphPad prism software. A heat map for FPRMS clone analysis was generated with dChip software (https://sites.google.com/site/dchipsoft/, accessed on 1 July 2021).

## 3. Results

### 3.1. Identification of Putative Cancer Stem Cell Subpopulations in FPRMS Cell Lines

**Figure 1:**
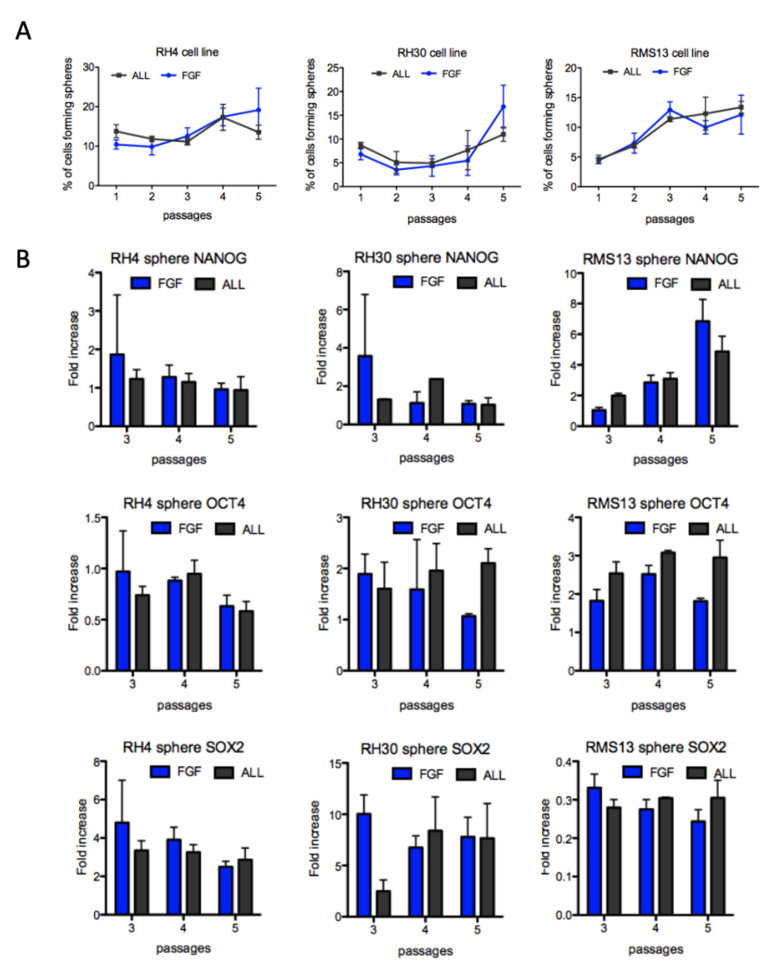
No enrichment of cancer stem-like cells in FPRMS spheres. (**A**) Sphere assay with RH4, RH30, and RMS13 cells cultivated in sphere medium supplemented with bFGF (FGF) or with bFGF, EGF, and PDGF (ALL) over indicated number of passages. Percentage of cells forming spheres was calculated at each passage. Represented are means of triplicates (*n* = 3). (**B**) Quantification of mRNA expression by real-time PCR of NANOG, OCT4, and SOX2 in spheres of three FPRMS cell lines at passage 3, 4, and 5. Fold increases are calculated compared to the cells cultured as a monolayer. Represented are means of triplicates (*n* = 3).

To understand whether FPRMS possess a subpopulation of CSCs, we developed a medium able to maintain the seven common FPRMS cell lines (RH4, RH30, RMS13, RH3, RH5, RH41, CW9019) in suspension, allowing the formation of spheres. Sphere medium was either supplemented with 10 ng/mL bFGF or with 10 ng/mL bFGF, EGF, and PDGF. While all FPRMS cells initially were able to form spheres, only three cell lines (RH4, RH30, and RMS13) showed self-renewal at clonal density and could be passaged for more than five weeks (Figure 1A and Figure S1A). However, the increase in the sphere forming cell fraction was very moderate over serial passages. Upregulation of the stem cell genes occurred only to a modest level but never in all the analyzed cell lines simultaneously. For instance, NANOG was upregulate in FGF and ALL media only in RMS13 spheres at passage 4 and 5 but not in the other cell lines (Figure 1B and Supplemental Figure S1B). SOX2 instead was strongly upregulated in both media in the cell lines RH4 and RH30 but was downregulated in RMS13, when compared to the monolayer cell culture (Figure 1B).

Finally, we also observed differences between the seven analyzed cell lines: only three of them (RH4, RH30 and RMS13) were capable of prolonged clonal passaging under these conditions. Thus, these data indicate that, in contrast to FNRMS [30], no enrichment of CSCs was observed in FPRMS using these sphere conditions.

**Figure 2:**
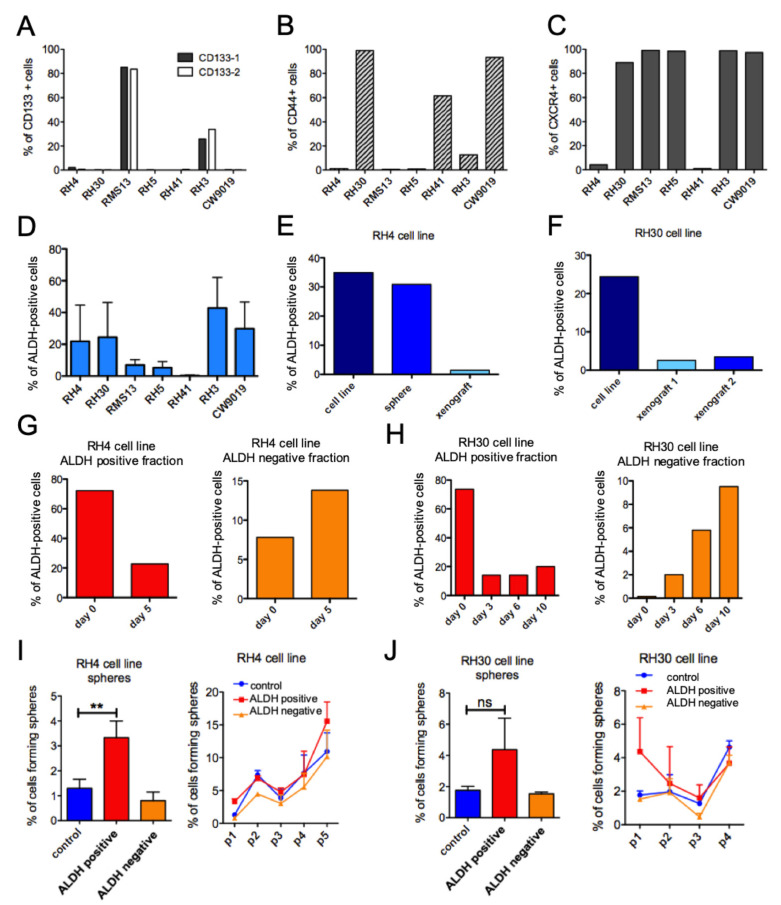
Lack of subpopulations with cancer stem-like properties in FPRMS cells lines. (**A**–**C**) Flow cytometric analysis of CSC markers CD133 (epitope 1 and epitope 2), CD44, and CXCR4 in seven FPRMS cell lines. Percentage of positive cells is indicated. (**D**) Percentage of ALDH-positive cells after staining with Aldefluor and flow cytometric analysis in FPRMS cell lines. (**E**,**F**) Percentage of ALDH-positive cells after staining with Aldefluor in RH4 (**E**) and RH30 (**F**). FPRMS cells cultured as a monolayer (cell line), or under sphere conditions, or cells dissociated from xenografts. G, H Distribution of sorted ALDH-positive and -negative fractions in RH4 (**G**) and RH30 (**H**) cell lines after indicated days of culture. (**I**,**J**) Sphere assay with RH4 and RH30 sorted ALDH-positive, -negative, and control fractions (control fractions were unstained cells processed through the FACS machine and collected without sorting for specific subpopulations). Cells were cultured at clonal density in ALL-sphere medium and primary sphere formation as well as self-renewal ability over 5 passages (p) was quantified. (unpaired *t*-test: ** *p* < 0.005, ns: not significant).

Next, we analyzed expression of three cell surface markers known to characterize different CSC subpopulations in mesenchymal tumors including sarcomas. For glycoprotein CD133 (PROMININ 1), characterizing FNRMS subpopulations [30], most of the FPRMS cell lines were found to be negative, except RMS13 and RH3, in which 30–90% of the cells stained positive (Figure 2A). CD44 is a major hyaluronan receptor shown to play a crucial role in the activation of tumor-promoting signaling pathways [38,39]. Four out of seven FPRMS cell lines possessed a subpopulation of positive cells, varying from 15% in RH3 to almost 100% in RH30 and CW9019 lines (Figure 2B). Three cell lines (RH4, RMS13, and RH5) were negative. Finally, CXCR4 expression was observed in close to 100% of the cells in all FPRMS cell lines except RH4 and RH41 (Figure 2C). Thus, despite the high heterogeneity between the seven most common FPRMS cell lines, none of the investigated potential CSC markers characterized a small subpopulation across the majority of the FPRMS cell lines and could therefore be used to enrich a putative stem-like subpopulation.

### 3.2. FPRMS Cell Lines Possess a Dynamic Subpopulation of Aldh Positive Cells Which Do Not Have Stem Cell Properties

To determine whether FPRMS possess cells with highly active ALDH, recently identified as a marker for CSC in many different tumors such as breast [40], ovary [41], lung [42], and sarcomas [43], we stained the same seven FPRMS cell lines with Aldefluor substrate. Indeed, we identified a subpopulation of ALDH-positive cells in all FPRMS lines, ranging from 3% in RH41 to about 40% in RH3 (Figure 2D). A small subpopulation was also seen in cells grown as spheres and in tumor xenografts in vivo. However, in vivo the subpopulation was lower (Figure 2E,F). To determine whether these ALDH-positive cells possessed increased stemness properties, the fractions in RH30 and RH4 cells were separated and their stability was assessed after five days for RH4 (Figure 2G) and three, six, and ten days for RH30 (Figure 2H). The ALDH-positive fraction generated ALDH-positive and -negative cells, as would be expected for cells showing stem-like properties. Surprisingly, in the ALDH-negative fraction a small population of ALDH-positive cells reappeared after three days of culture, which further increased after 10 days. Thus, cells sorted on ALDH activity do not represent a stable but rather a dynamic population. Finally, when tested for sphere formation, and no statistically significant difference was observed between the fractions and no enrichment over time was observed (Figure 2I,J). These populations also showed no significant upregulation of the core stem cell genes by gene expression analysis in the ALDH-positive fraction but rather a slight decrease in NANOG expression (Figure S2A,B). In conclusion, FPRMS cell lines possess a subpopulation of cells with high ALDH activity but these cells do not display increased and stable stem-like properties.

Stem cells in adult organs are usually more quiescent compared to the transit-amplifying progenitor cells [44]. In order to investigate also whether FPRMS contain a population of quiescent stem cells, we stained RH4 and RH30 cells with the DNA dye PKH26 and followed dye dilution at different time points by flow cytometry (Figure S3A,B). One day after staining both cell lines showed more than 98% of PKH26^high^ cells, which diminished after three days to 92% PKH26^high^ for RH4 and 80% PKH26^high^ for RH30 cells. However, after 14 days in culture no PKH26^high^ cells were detectable anymore in cell lines. Thus, although the two cell lines differed in their proliferation rate, no subpopulation of slow-proliferating cells was observed.

### 3.3. PAX3-FOXO1 but Not the Stem Cell Regulators NANOG, OCT4, and SOX2 Affects Cellular Physiology

**Figure 3:**
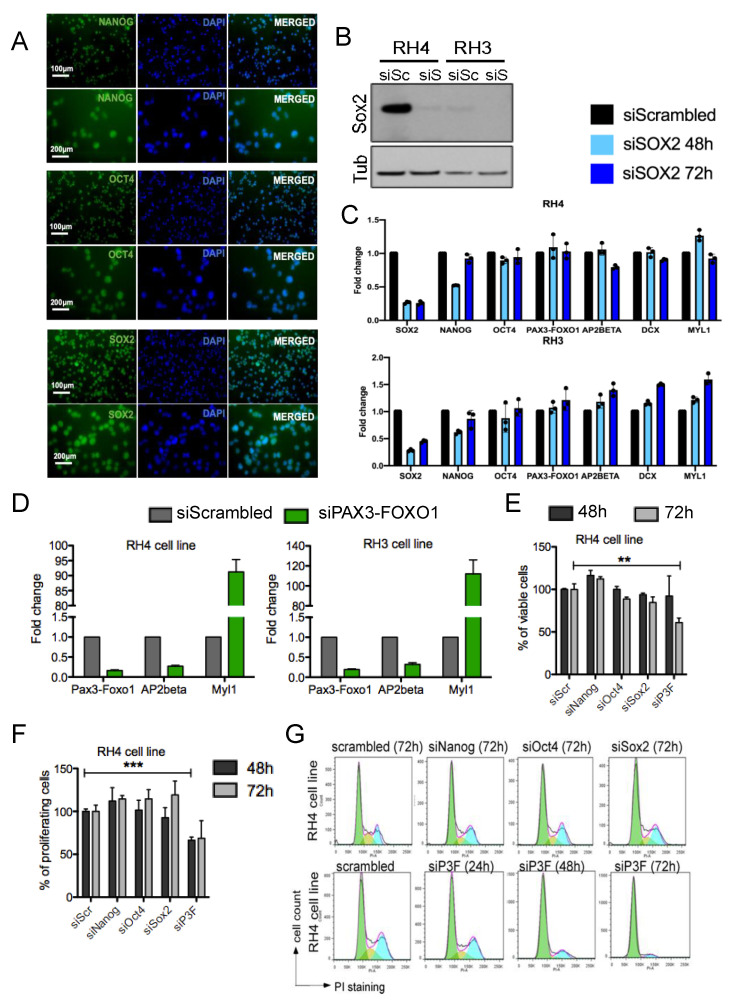
FPRMS cells express the core stem cell factors but only altered expression of PAX3-FOXO1 affects their cellular physiology. (**A**) Immunofluorescence for NANOG, OCT4, and SOX2 in RH3 cell line. (**B**) Western blot analysis of SOX2 expression after 72 h of siRNA-mediated silencing in RH4 and RH3 FPRMS cells. siSc = siScrambled; siS = siSOX2. (**C**) RNA expression analysis for the indicated genes after 48 h and 72 h of SOX2 silencing with a specific siRNA in RH4 and RH3 cells. Fold changes are calculated compared to cells treated with scrambled siRNA. Represented are means of triplicates (*n* = 3). (**D**) RNA expression analysis for the indicated genes after 72 h silencing of PAX3-FOXO1 in RH4 and RH3 cells. Fold changes are calculated compared to cells treated with scrambled siRNA. Represented are means of triplicates (*n* = 3). (**E**) Cell viability measurements (WST-1 assay) upon 48 h and 72 h of knockdown of PAX3-FOXO1 (siP3F), NANOG, OCT4, and SOX2 in RH4 cells. Percentage of viable cells was calculated compared to scrambled cells (siScr). Represented are means of triplicates (*n* = 3). Statistical analysis: Unpaired *t*-test, ** *p* < 0.001. (**F**) Cell proliferation assay (BrdU incorporation) after 48 h and 72 h of PAX3-FOXO1, NANOG, OCT4, and SOX2 knockdown in RH4 cells. Percentages are calculated relative to the scrambled control cells. Represented are means of triplicates (*n* = 3). Statistical analysis: Unpaired *t*-test, *** *p* < 0.0001. (**G**) Flow cytometric analysis of RH4 cell cycle distribution using propidium iodide (PI) upon silencing of PAX3-FOXO1 (siP3F), NANOG, OCT4, and SOX2 for the indicated time.

The transcription factors NANOG, OCT4, and SOX2 are key regulators of pluripotency and play crucial roles in the maintenance of CSCS in many different tumors [27,32,45,46]. Expression analysis in the seven FPRMS cell lines by immunohistochemistry and gene expression analysis revealed that all cell lines expressed these genes in the majority of the cells (Figure 3A and Figure S4A–C). To understand their possible function in FPRMS, we performed siRNA-mediated silencing in RH4 and RH3 cells and measured expression of a set of FPRMS associated genes, cell proliferation, and viability, and cell cycle distribution. Surprisingly, knockdown of the pluripotency factors did not appear to impact FPRMS cells on either a molecular or functional level. Depletion of SOX2 with specific siRNA sequence (Figure 3B) did not upregulate differentiation markers such as MYL1 or induce differential gene expression of PAX3-FOXO1 and its target gene AP2β after 48 and 72 h (Figure 3C). Similar results were obtained upon NANOG and OCT4 knockdown. In contrast, depletion of PAX3-FOXO1 induced downregulation of its target genes and strong upregulation of muscle differentiation factors (Figure 3D). Cell viability and proliferation was significantly altered only upon downregulation of the fusion protein (Figure 3E,F). Finally, cell cycle distribution was unaltered by depletion of the stem cell genes whereas cells arrested at the G1 cell cycle phase after PAX3-FOXO1 silencing (Figure 3G).

**Figure 4:**
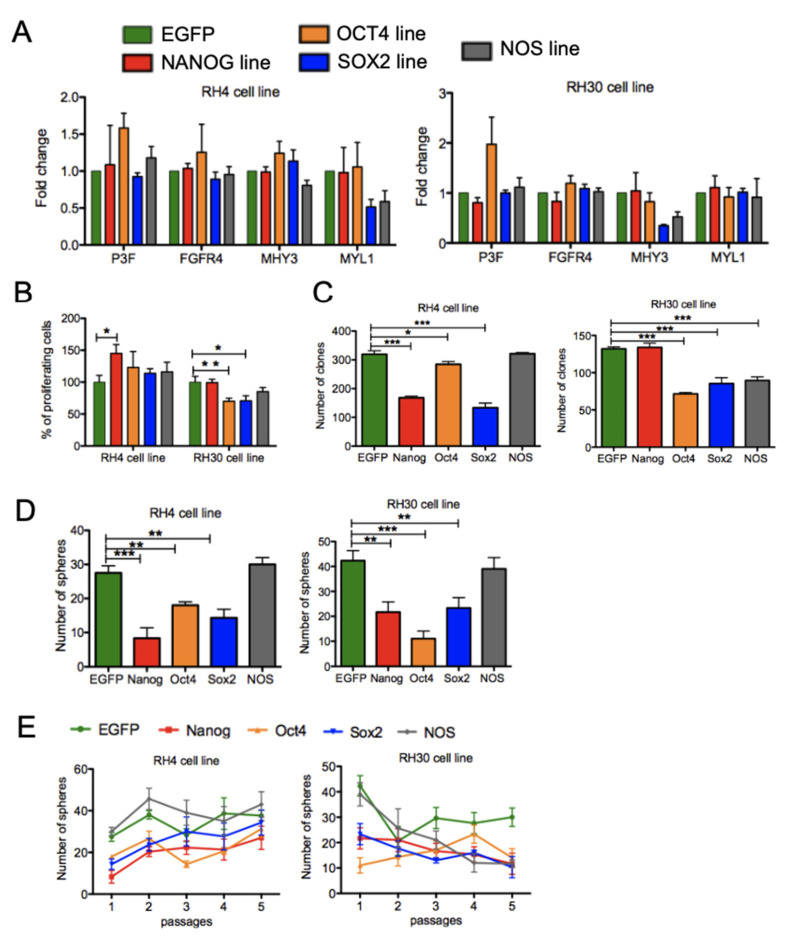
Overexpression of the core stem cell factors does not enhance stem cell properties. (**A**) RNA expression analysis Figure 4. and RH30 transgenic cell lines overexpressing the core stem cell genes NANOG, OCT4, and SOX2 singularly or simultaneously (NOS line). For each FPRMS line an EGFP transgenic cell line was generated and used as control line. Fold changes are calculated compared to the EGFP lines. (**B**) Cell proliferation assay (BrdU incorporation) in RH4 and RH30 transgenic cell lines. Percentages are calculated relative to EGFP control line. Represented are means of triplicates (*n* = 3). Statistical analysis: Unpaired *t*-test, ** *p* < 0.005, * *p* < 0.05. (**C**) Clonogenic assay with RH4 and RH30 transgenic cell lines. Clones were stained with crystal violet and quantified. Represented are means of triplicates (*n* = 3). Statistical analysis: Unpaired *t*-test, *** *p* < 0.0005, * *p* < 0.05. (**D**) Primary sphere formation with RH4 and RH30 transgenic cell lines. Represented are means of triplicates (*n* = 3). Statistical analysis: Unpaired *t*-test, *** *p* < 0.0005, ** *p* < 0.005. (**E**) Sphere assay with RH4 and RH30 lines overexpressing the stem cell genes.

To further validate these observations, we generated stable transgenic RH30 and RH4 cells overexpressing the stem cell genes (Figure S5), either singly or simultaneously. An EGFP overexpressing cell line was also generated and used as a control. These cells did not consistently alter differentiation as tested by MHY3 and MYL1 expression (Figure 4A), nor did they show altered number of proliferating cells (Figure 4B). Furthermore, RH4 and RH30 transgenic cell lines showed a reduced number of clones in comparison to the control line. Surprisingly, cells overexpressing NANOG, OCT4, and SOX2 had a reduced ability to form primary spheres (Figure 4D) and to self-renew over a period of five passages (Figure 4E).

These data confirm earlier observations that PAX3-FOXO1 expression is essential for FPRMS cell maintenance whereas interestingly the expression of the core stem cell genes in FPRMS cells is dispensable and not sufficient to alter cellular physiology. Although further analysis would be necessary to fully understand the role of the core stem cell genes in FPRMS cells, our data reinforce the hypothesis that the fusion protein, and not an exclusive subpopulation of CSCs, is the essential factor controlling self-renewal and maintenance of FPRMS cells.

### 3.4. High Frequency of Tumor Initiating Cells in FPRMS

**Figure 5:**
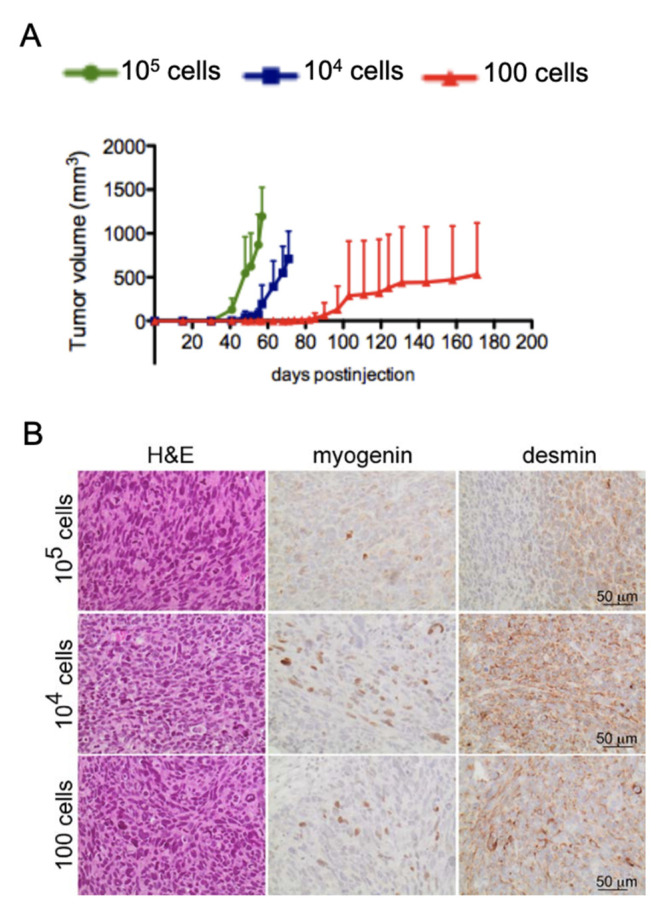
High frequency of tumor initiating cells in FPRMS cells. (**A**) In vivo limiting dilution with RH30 cells; 10^5^ (*n* = 3), 10^4^ (*n* = 4), and 100 cells (*n* = 5) were injected intramuscularly (i.m.) into the left leg of NOD/Scid mice. Tumor growth was monitored over a period of 180 days. (**B**) Immunohistochemical analysis of xenografts generated from RH30 cells after injection of 10^5^, 10^4^, and 100 cells. Tumors were stained with H&E and the FPRMS markers MYOGENIN and DESMIN.

Since we did not find evidence for an exclusive CSC subpopulation in FPRMS, we next performed in vivo limiting dilutions with RH30 cells to directly assess their tumor initiating potential. Monolayer cultures of RH30 cells were collected and three different dilutions of cells (10^5^, 10^4^, and 100 cells) were injected intramuscularly into the left leg of NOD/Scid mice. All mice injected with the two higher cell dilutions (10^5^, *n* = 3 and 10^4^, *n* = 4) developed tumors and three out of five mice injected with only 100 cells showed tumor growth starting 80 days post-injection (Figure 5A). Immunohistochemical analysis of the xenografts confirmed their FPRMS diagnosis, showing typical small blue round cell morphology and expression of the myogenic markers MYOGENIN and DESMIN (Figure 5B). These data, together with the fact that we did not find a subpopulation of CSCs using the classical in vitro assays, indicated that the frequency of cells with tumor initiating capacity in the FPRMS tumor might be high.

**Figure 6:**
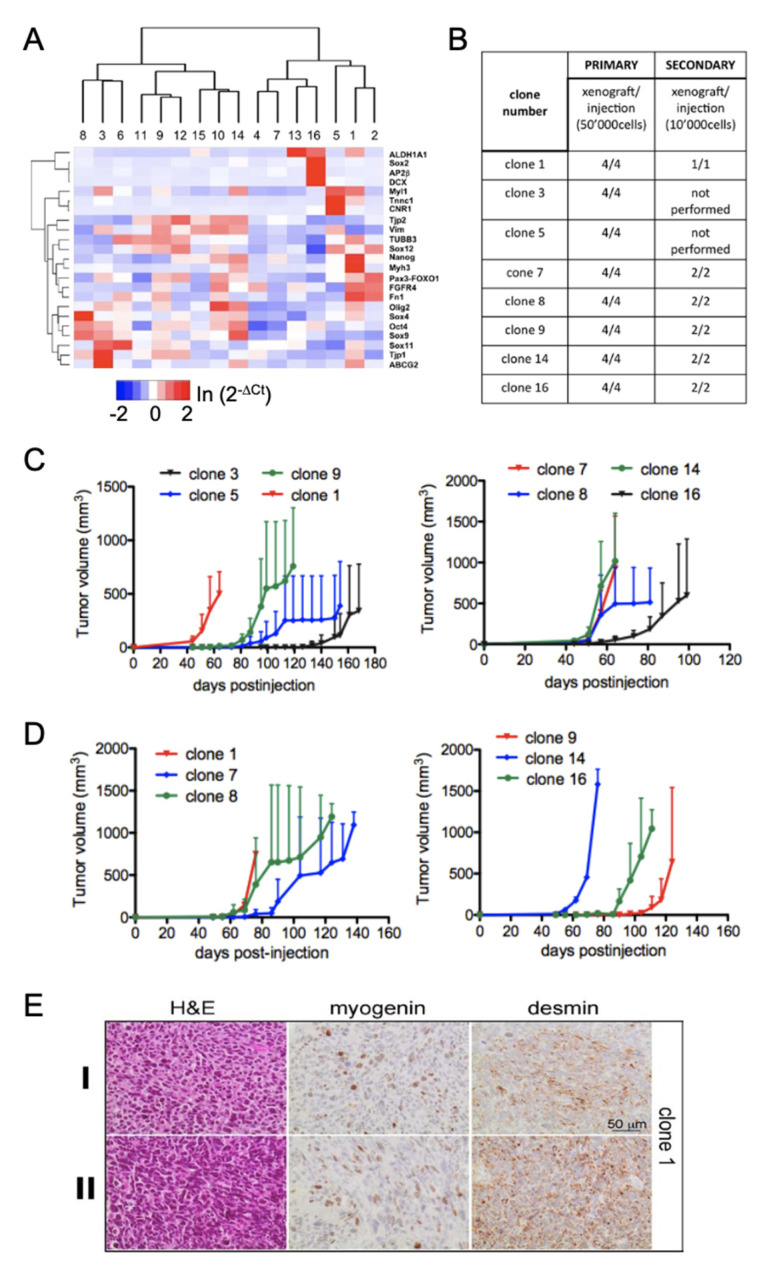
Clonal analysis of cells. (**A**) Heat map of qRT-PCR of total RNA relative to GAPDH from RH30 single-cell derived clones. Sixteen clones were analyzed for the expression of the genes indicated. Scale bar ln(2^−^^ΔCt^). (**B**) Generation of primary and secondary xenografts with 8 selected RH30 clones. For primary xenograft generation 5 × 10^4^ cells were injected i.m into the left leg of NOD/Scid mice (*n* = 4). Single cell suspensions (10^4^ cells) of primary tumors were injected i.m into mice (*n* = 2) for secondary tumor formation. (**C**) Tumor growth of primary xenograft clones generated from one RH30 cell. (**D**) Tumor growth of secondary xenografts. (**E**) Immunohistochemical staining of primary (I) and secondary (II) xenograft sections of clone 1. Tumors were stained with hematoxylin and eosin (H&E) and the FPRMS markers MYOGENIN and DESMIN.

To further assess whether in fact all cells might possess tumor initiation potential, we performed clonal analysis in vitro. RH30 cells expressing EGFP as marker were subcloned and sixteen single-cell derived clones were expanded. Gene expression analysis by qRT-PCR revealed that levels of PAX3-FOXO1, several target genes, stem cell factors as well as differentiation markers were differentially expressed indicating significant heterogeneity between the clones (Figure 6A). We selected eight clones with divergent gene expression and injected 5 × 10^4^ cells for each clone i.m. in mice as before (*n* = 4). Indeed, after 40 to 140 days post-injection all mice developed tumors with FPRMS histology (Figure 6B–D and Figure S6). To demonstrate directly that each clone had the capability to self-renew and propagate tumors, six primary tumors were used for secondary xenografts. For each clone, injection of 1 × 10^4^ primary xenograft cells was enough to generate secondary tumors (Figure 6B). Immunohistochemical analysis of primary and secondary xenografts confirmed FPRMS histology with variable expression of MYOGENIN and DESMIN (Figure 6E and Figure S6), attesting their heterogeneous origin.

In conclusion, these data demonstrate that similarly to the primary patient tumors a high intra-cellular heterogeneity is also observed in the FPRMS cell lines. Moreover, in vivo and in vitro limiting dilution experiments confirmed a high frequency of tumor initiating cells in the FPRMS line RH30.

## 4. Discussion

It has already been reported that fusion oncogene PAX3-FOXO1 maintains FPRMS cell survival [19,20,21], and hence its expression and transcriptional activity is of crucial importance. Here, we provide further evidence for its indispensable oncogenic role by demonstrating that FPRMS lack an exclusive cell subpopulation with higher tumorigenic potential, and also forced expression of the classical stem cell factors does not influence cell self-renewal. Rather, all cells possess tumorigenic potential and their maintenance depends on the presence of the fusion protein.

Functional tumor heterogeneity is an important determinant of therapy resistance as recently shown by clonal analysis for myeloid and lymphoblastic leukemia [47,48]. We previously identified a small subpopulation of cells with stem-like potential in fusion FNRMS by a functional sphere assay [30]. In this case, sphere cells were positive for the CSC marker CD133, expressed the core stem cell factors, and showed enhanced tumorigenic potential in vivo.

In this present study, we developed new culture conditions able to maintain spheres generated with most of the available FPRMS cell lines. Two sphere media were tested, consisting of DMEM/HAM’s F12 supplemented with KSR, 10 ng/mL bFGF or with 10 ng/mL bFGF, EGF, and PDGF. Interestingly, no differences were observed in the ability of forming spheres in all seven analyzed FPRMS cell lines when the sphere media was supplemented with only bFGF or with all three growth factors. Although we could maintain three out of seven FPRMS lines for five weeks under the new established sphere conditions, the observed increase in sphere numbers as well as the upregulation of the core stem cell genes compared to the monolayer cell culture was very moderate and not consistent for all the analyzed cell lines. Recently, Slemmons et al. established other sphere culture conditions able to maintain two FPRMS cell lines in suspension [49]. Although the spheres were not passaged at clonal level, they showed upregulation of NANOG, OCT4, and SOX2 after serial passaging when compared to the monolayer culture, but not with xenograft derived cells. The sphere medium used by Slemmons et al. contained eight times more bFGF and four times more EGF than the medium used in our study. Instead of PDGF they added insulin to the neurobasal media supplemented with B27 [49]. We consciously used the minimal concentration of growth factors allowing passaging of the spheres in order to exclude a media-dependent induced cell plasticity. The media established by Slemmons et al. is based on the media developed in our laboratory for culturing FNRMS spheres [30]. Moreover, our spheres were passaged at clonal density for several passages without showing upregulation of the stem cell genes. Slemmons et al. did not passage the spheres at clonal density, but regularly split them one to two. The authors further showed that under these conditions human skeletal muscle myoblasts were not induced to form spheres and the cells died, concluding that no cellular reprogramming happened due to the culture conditions.

Only ALDH activity distinguished a small subpopulation of positive cells in all FPRMS cell lines analyzed. Nevertheless, these cells were not stable and did not show enhanced stem cell properties when compared to the negative fraction. Similarly, CD133 expression was noted in FPRMS before but did not alter during the course of the disease [50]. Therefore, we hypothesized that FPRMS, contrary to FNRMS but similarly to acute lymphoblastic leukemia and melanoma [48,51], possess a high abundance of cells with tumor initiating potential. Corroborating this hypothesis, expression of the pluripotency genes NANOG, OCT4, and SOX2 was homogeneous in all analyzed monolayer cultures of FPRMS cell lines, as also confirmed by Slemmons et al. [49].

NANOG, OCT4, and SOX2 mainly promote tumorigenesis by regulating the CSCs [28,32], and therefore high expression of these stem cell factors is associated with poorer outcomes in numerous cancers [52,53,54]. For example, NANOG was determined to be important for FNRMS self-renewal, possibly acting downstream of hedgehog signaling [55]. The fact that neither silencing of the pluripotency factors nor their overexpression in FPRMS cells influenced stemness properties, whereas downregulation of PAX3-FOXO1 immediately resulted in a strong upregulation of muscle differentiation factors, cell cycle arrest, and subsequent apoptosis, underline their secondary role in FPRMS. Thus, the fusion gene PAX3-FOXO1 and not the stem cell factors might be the key regulator in FPRMS. It is likely that fusion protein-specific target gene expression might contribute to this function, however the responsible target gene(s) remain unclear at the moment and the underlying molecular mechanisms need to be further characterized. Epigenetic factors might play a fundamental role in maintaining stem-like properties, and JARID2 and EZH2 have indeed been described as PAX3-FOXO1 target genes [23], however their influence on stem-like properties has not been investigated. Alternatively, PAX3-FOXO1 was recently shown to be dynamically regulated during the cell cycle, being higher expressed in the G2 phase where it acts in G2/M checkpoint adaptation, leading to cell cycle progression instead of programmed cell death [56].

Whether high frequency of tumor initiating stem-like cells is a characteristic common to most tumors bearing chimeric transcription factors remains also to be elucidated. For example, results similar to the ones presented here were recently published for synovial sarcoma, which expresses the chimeric oncogene SS18-SSX [57]. There, despite high expression of the pluripotency markers NANOG and OCT4, cells differentiated only upon depletion of the fusion protein. Therefore, this tumor has been regarded as stem cell malignancy being mainly driven by the fusion protein [57]. On the other hand, the role of EWS-FLI1 in Ewing sarcoma is less clear. While the fusion protein was found to modulate SOX2 expression [58], a CD133 subpopulation with cancer stem cell properties was identified [33]. Recently, Franzetti et al. showed that EWS-FLI1-high and -low cells differ in their physiological properties: high cells rather proliferate while low cells rather migrate and metastasize. [59] A similar speculation is found for PAX3-FOXO1 while using murine cells [60].

Our study was not able to identify specific subpopulations with stem cell properties in seven FPRMS cell lines, but we could clearly demonstrate with the clonal analysis in vivo and in vitro high frequency of tumor initiating cell and the indispensable role of PAX3-FOXO1 in maintaining the identity of these cells. With the recently developed media for FPRMS sphere culture, it would be interesting to investigate the fusion protein in spheres and in primary PDX cultures [61].

## 5. Conclusions

In conclusion, our results demonstrate that FPRMS and FNRMS [30] differ fundamentally in respect to their cellular organization, and hence will likely require different treatment approaches. Moreover, we identified a novel physiological role for the PAX3-FOXO1 fusion oncoprotein, which reinforces the importance of the fusion protein as a therapeutic target for this pediatric sarcoma. The high frequency of tumor propagating cells in FPRMS could explain their higher propensity for metastasis and relapse. Although the role of the stem cell genes in the FPRMS cells need to be further investigated, the use of cell lines instead of primary tumors represents a clear limitation. Further analysis would be needed and the use of primary patient derived xenografts could represent a suitable tool, since they closely resemble the original tumor [61].

## Supplementary Materials

The following are available online at https://www.mdpi.com/article/10.3390/genes12091373/s1. Table S1: TaqMan® Gene Expression Assays (Life Technologies). Table S2: Antibodies list. Figure S1: No enrichment of cancer stem-like cells in rhabdospheres from several FPRMS cell lines. Figure S2: High ALDH activity does not characterize FPRMS cancer stem-like cells. Figure S3: No asymmetrical cell division in FPRMS cell lines. Figure S4: FPRMS cell lines homogenously express low levels of the core stem cell genes. Figure S5: Western blot analysis for RH4 and RH30 transgenic cell lines. Figure 6: Immunohistochemical staining of RH30 xenografts.
