# Supplementary material for: High Frequency of Tumor Propagating Cells in Fusion-Positive Rhabdomyosarcoma
Source: Genes (Basel). 2021 Aug 31;12(9):1373. doi: 10.3390/genes12091373 (PMC8469567; doi:10.3390/genes12091373)

Supplementary Material and Methods

Supplemental Table S1: TaqMan® Gene Expression Assays (Life Technologies)

**Gene name**	**TaqMan assay number**
ALDH1A1	Hs00946916_m1
TFAP2B (AP2B)	Hs01560931_m1
FGFR4	Hs01106908_m1
GAPDH	Hs02758991_g1
GATA4	Hs00171403_m1
MYH3	Hs01074230_m1
MYL1	Hs00984899_m1
NANOG	Hs02387400_g1
OLIG2	Hs00300164_s1
PAX3-FOXO1	Hs03024825_ft
POU5F1 (OCT4)	Hs04260367_gH
Sox2	Hs04234836_s1
TNNC1	Hs00896999_g1

Supplemental Table S2: Antibodies list

Primary antibody (clone)	Company	Application (dilution)
mouse anti-NANOG (hNanog.2)	eBioscience	WB (1:500), IF (1:50)
rabbit anti-OCT4 (C52G3)	Cell Signaling	WB (1:500), IF (1:50)
rabbit anti-SOX2 (D6D9)	Cell Signaling	WB (1:500), IF (1:50)
mouse anti-human Desmin (D33)	DAKO	IHC (1:20)
mouse anti-Myf4 (L026)	Novocastra Laboratories Ltd	IHC (1:20)
rabbit anti-mib1 (30-9)	Ventana-Roche	-
phalloidin-FITC	Enzo	IF (1:2000)
**Secondary antibody**	**Company**	**Application (dilution)**
anti-mouse HRP-linked 7076S	Cell Signaling	WB (1:2000)
anti-rabbit HRP-linked 7074S	Cell Signaling	WB (1:2000)
anti rabbit Alexa Fluor 594	Invitrogen	IF (1:500)
anti mouse Alexa Fluor 594	Invitrogen	IF (1:500)

WB: Western Blotting; IF: Immunofluorescence; IHC: Immunohistochemistry

Supplemental Figure S1

No enrichment of cancer stem-like cells in rhabdospheres from several FPRMS cell lines
A Sphere assay with RH5, RH41, RH3, and the PAX7-FOXO1 positive CW9019 lines cultivated in sphere medium supplemented with bFGF (FGF) or with bFGF, EGF, and PDGF (ALL) over several passages. Percentage of cells forming spheres was calculated at each passage. The cell lines RH3 and CW9019 could be propagated for only 2 passages. **B** Total RNA expression of NANOG, OCT4, and SOX2 in spheres of three cell lines at different passages compared to the cells cultured as a monolayer.

Supplemental Figure S2

High ALDH activity does not characterize FPRMS cancer stem-like cells
A, B qRT-PCR expression analysis of sorted ALDH-positive cells compared to sorted ALDH-negative cells for the indicated genes in RH4 (**A**) and RH30 (**B**) cells.

Supplemental Figure S3

No asymmetrical cell division in FPRMS cell lines
A, B PKH26 staining of RH4 (**A**) and RH30 (**B**) FPRMS cell lines and flow cytometry analysis over a period of 14 days.

Supplemental Figure S4

FPRMS cell lines homogeneously express low levels of the core stem cell genes
A, B, C Immunofluorescence analysis in seven cell lines for the stem cell genes NANOG, OCT4, and SOX2 (upper panel). Basal level RNA expression relative to GAPDH for the stem cell genes in the indicated cell lines (lower panel).

Supplemental Figure S5

A Western blot analysis for RH4 and RH30 cell lines stably overexpressing NANOG, OCT4, SOX2, or the three genes simultaneously (NOS). The embryonic carcinoma line NTERA-2 was used as a positive control. NTERA-2 was maintained in DMEM supplemented with 10% FBS, 2 mM L-glutamine and 100 U/mL penicillin/streptomycin. (GFP = EGFP cell line, N = NANOG overexpressing cell line, O = OCT4 overexpressing cell line, S = SOX2 overexpressing cell line, NT2 = NTERA-2).

Supplemental Figure S6

Immunohistochemical staining of primary (I) and secondary (II) xenograft sections of three RH30 clones. Tumors were stained with hematoxylin and eosin (H&E) and the FPRMS markers MYOGENIN and DESMIN.

Supplemental FIGURE S1

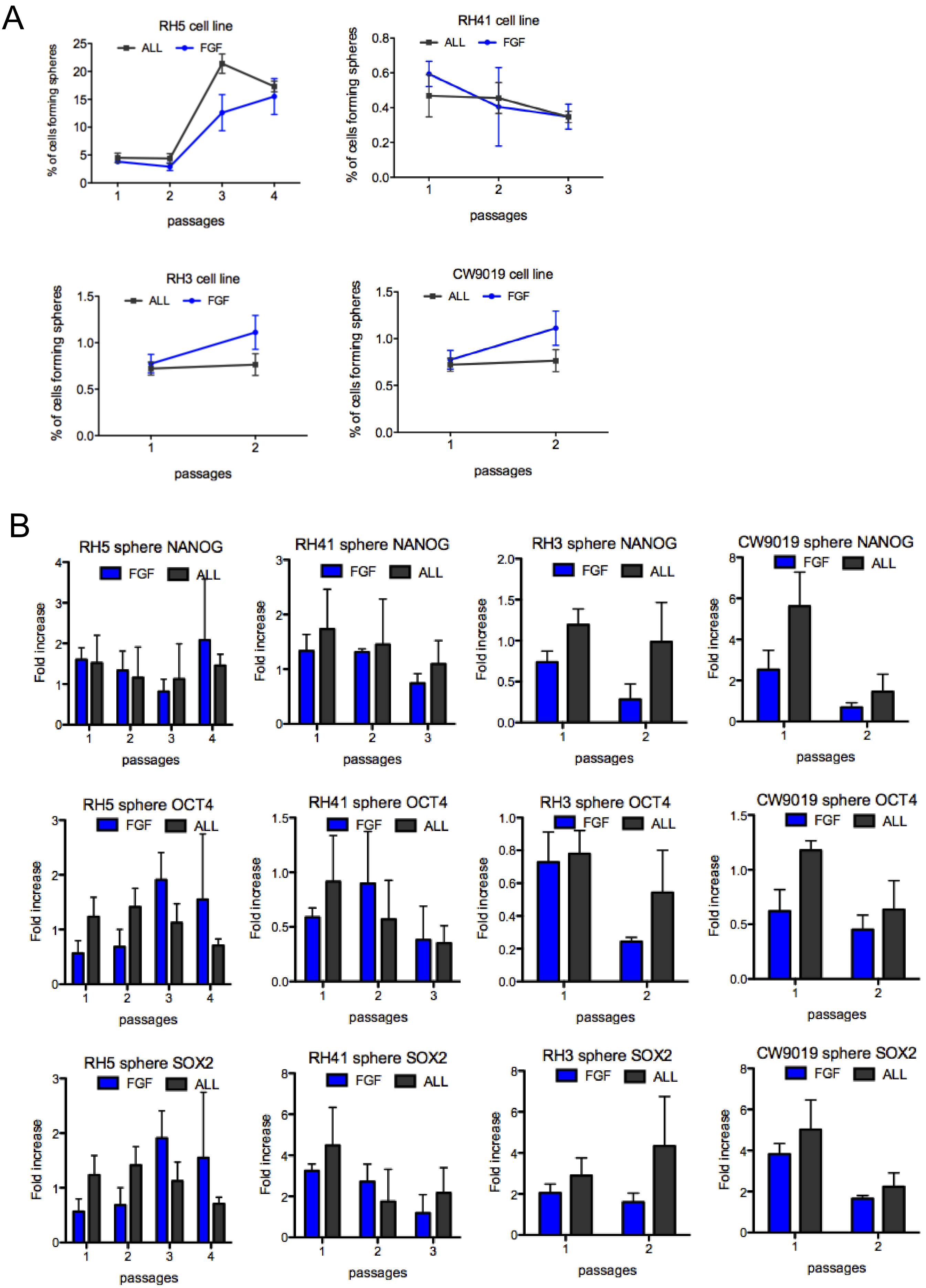

Supplemental FIGURE S2

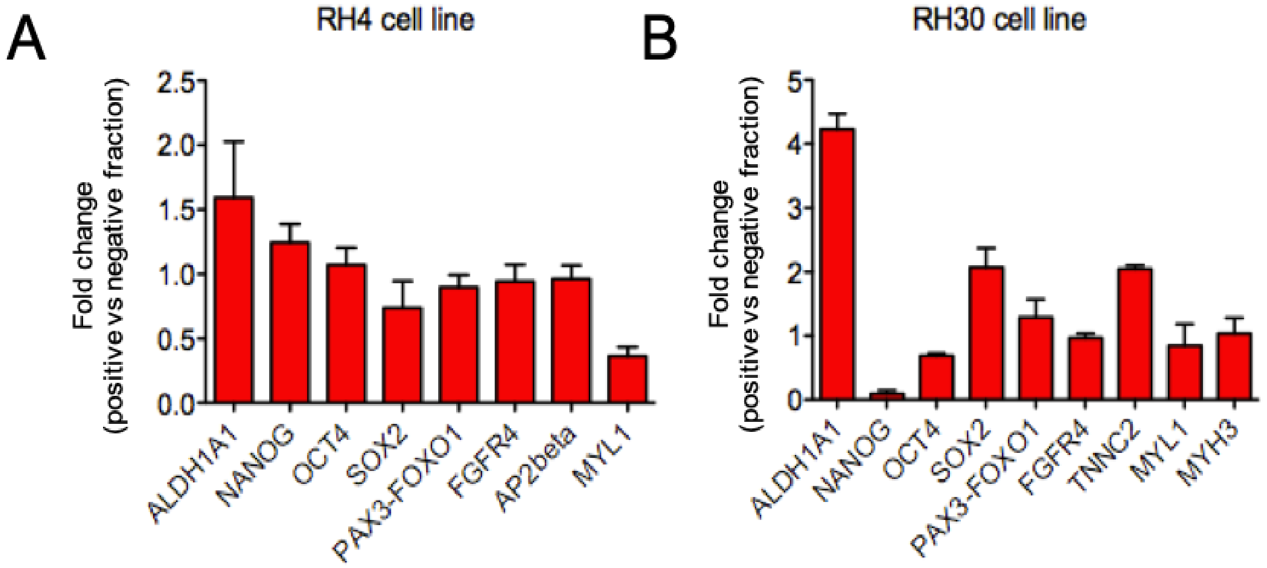

Supplemental FIGURE S3

A

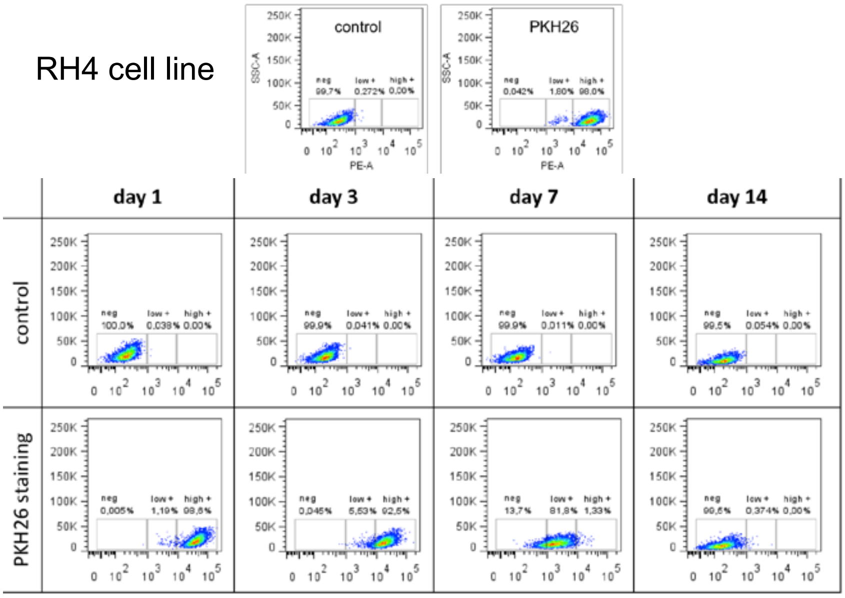

B

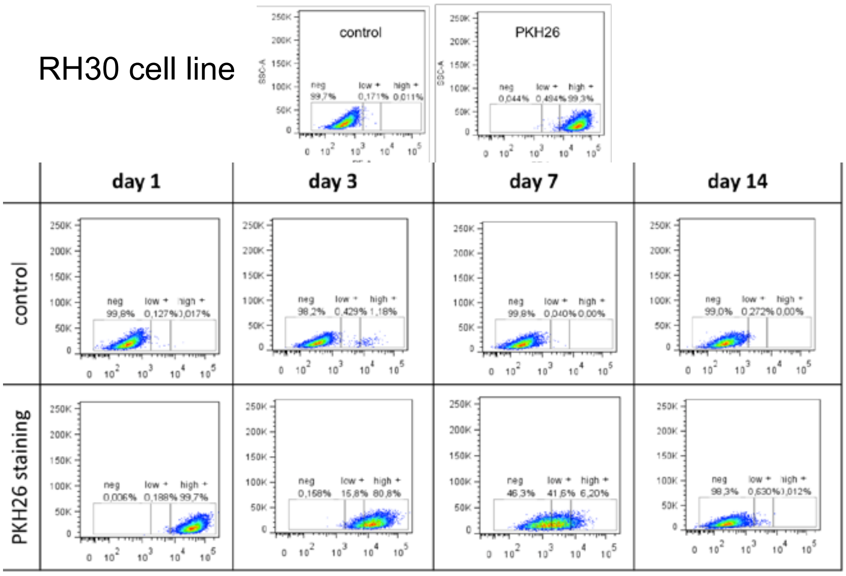

Supplemental FIGURE S4A

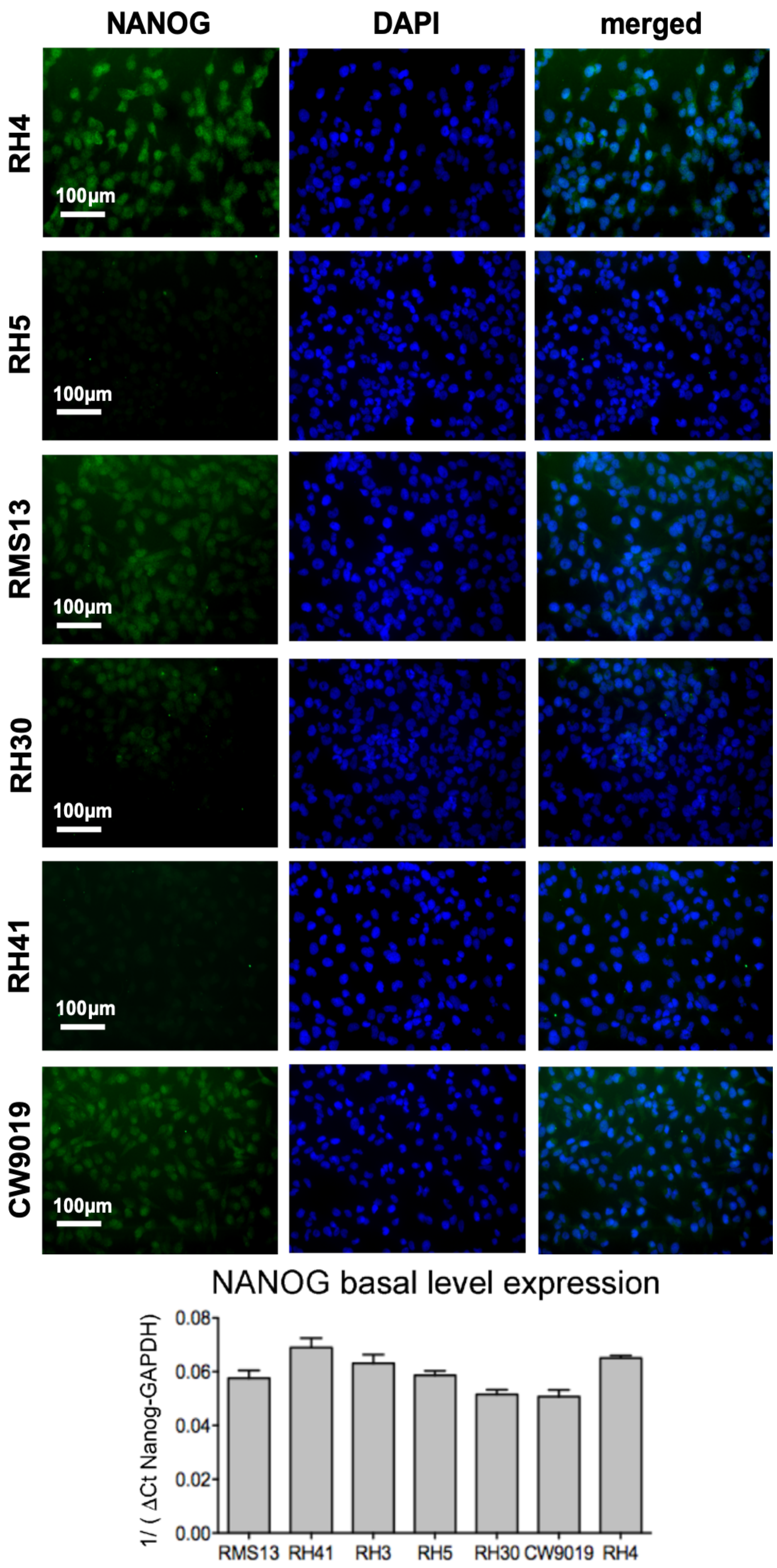

Supplemental FIGURE S4B

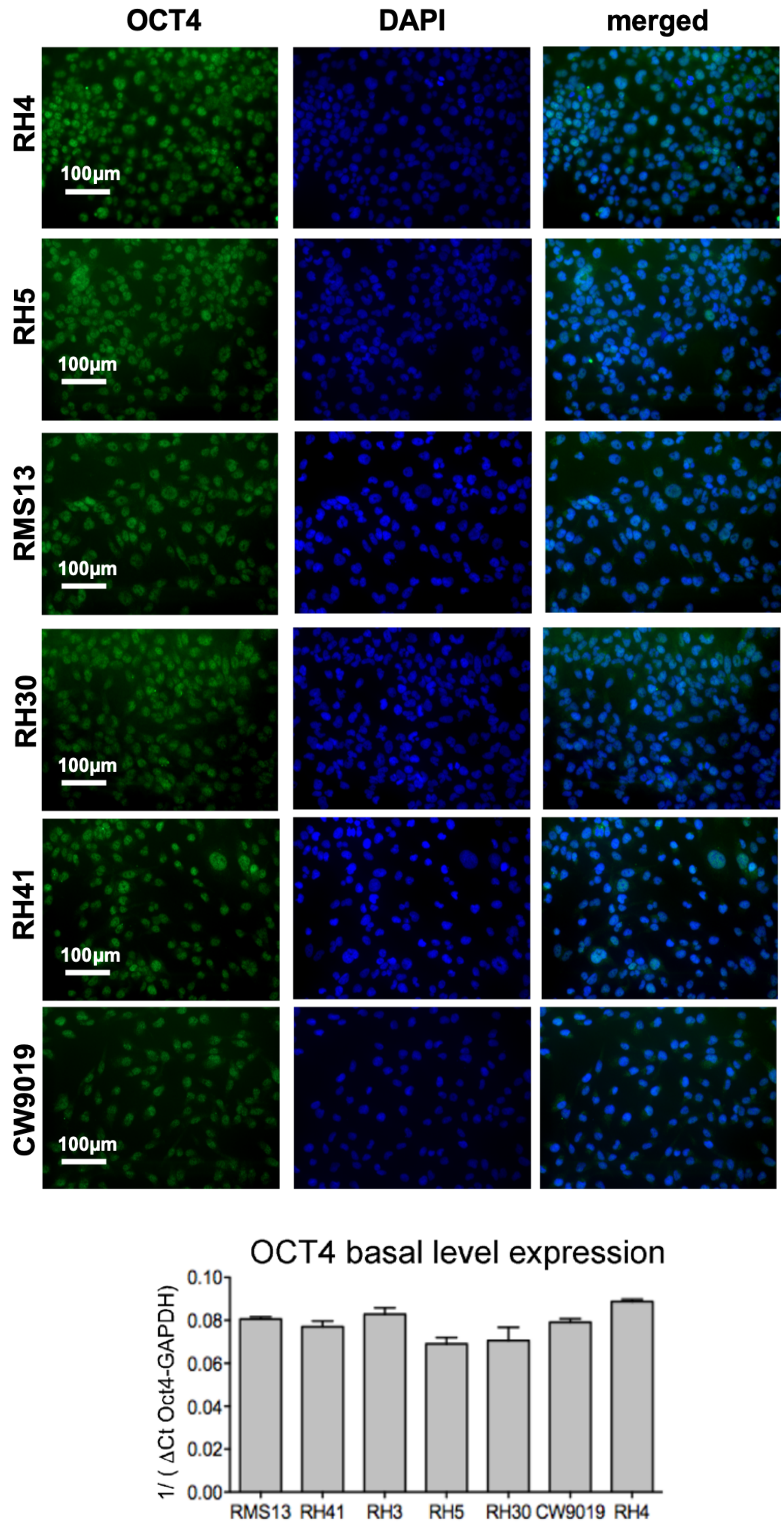

Supplemental FIGURE S4C

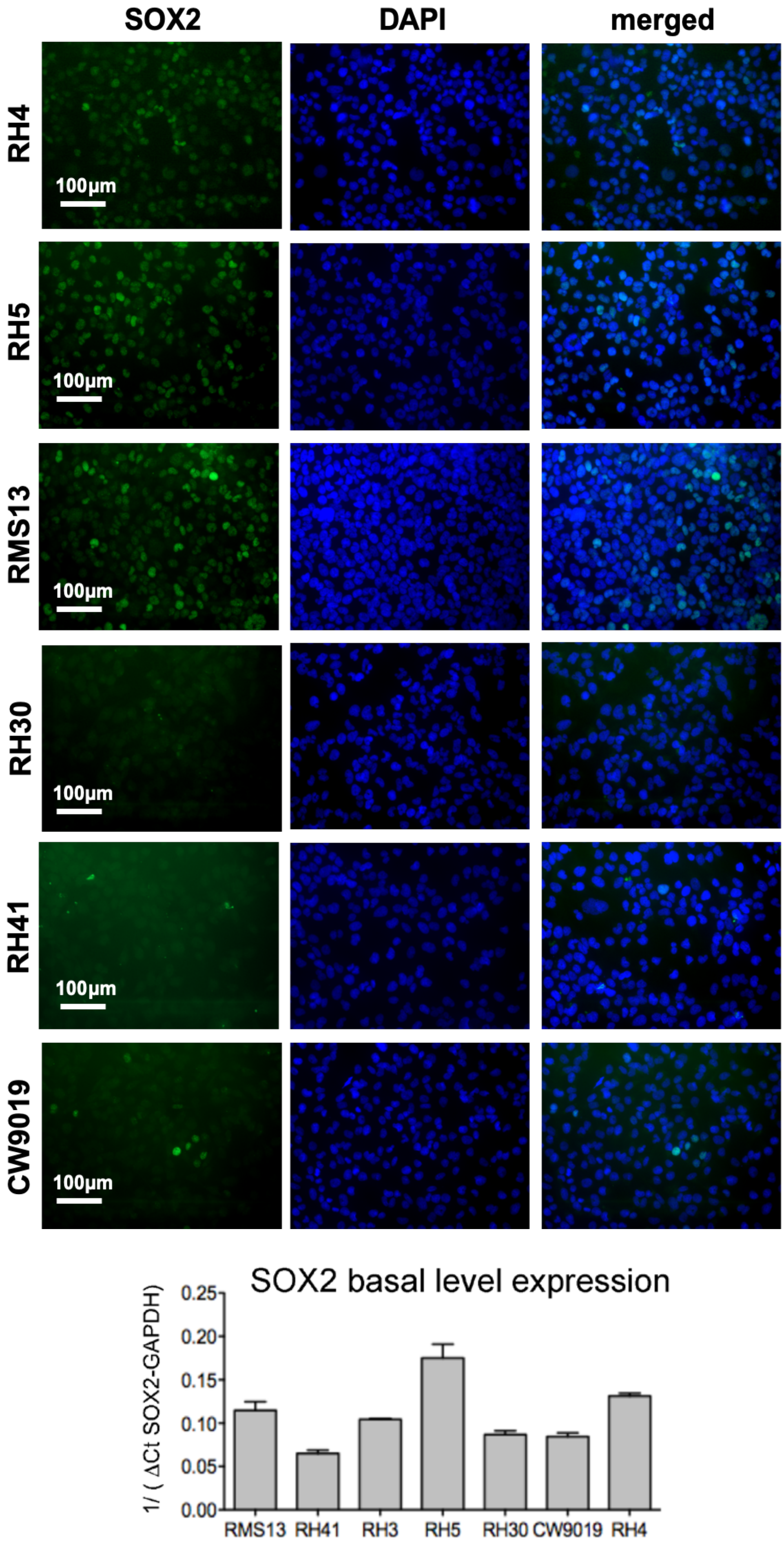

Supplemental FIGURE S5

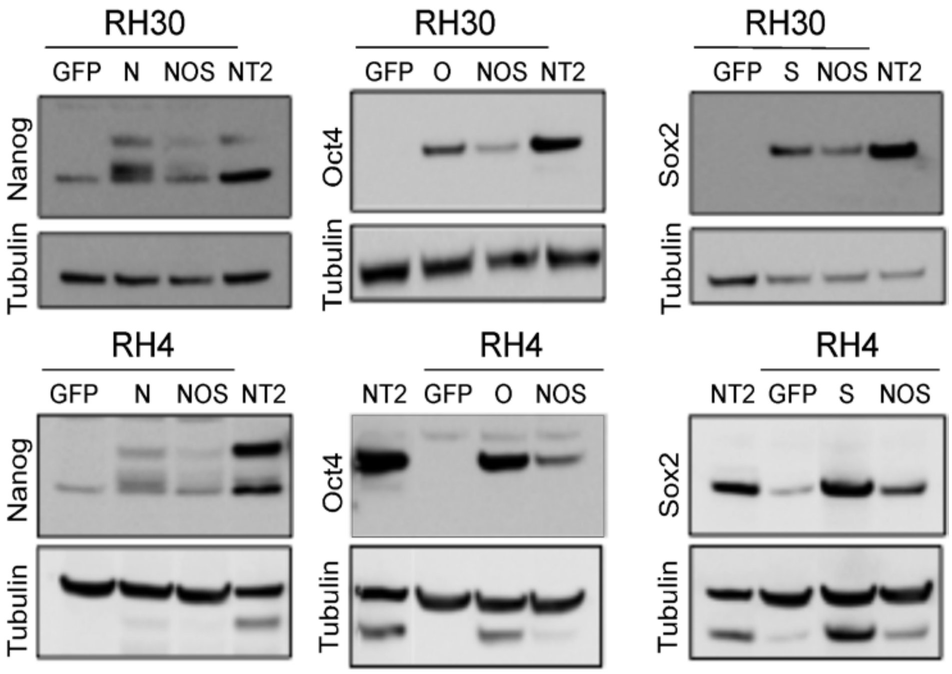

Supplemental FIGURE S6

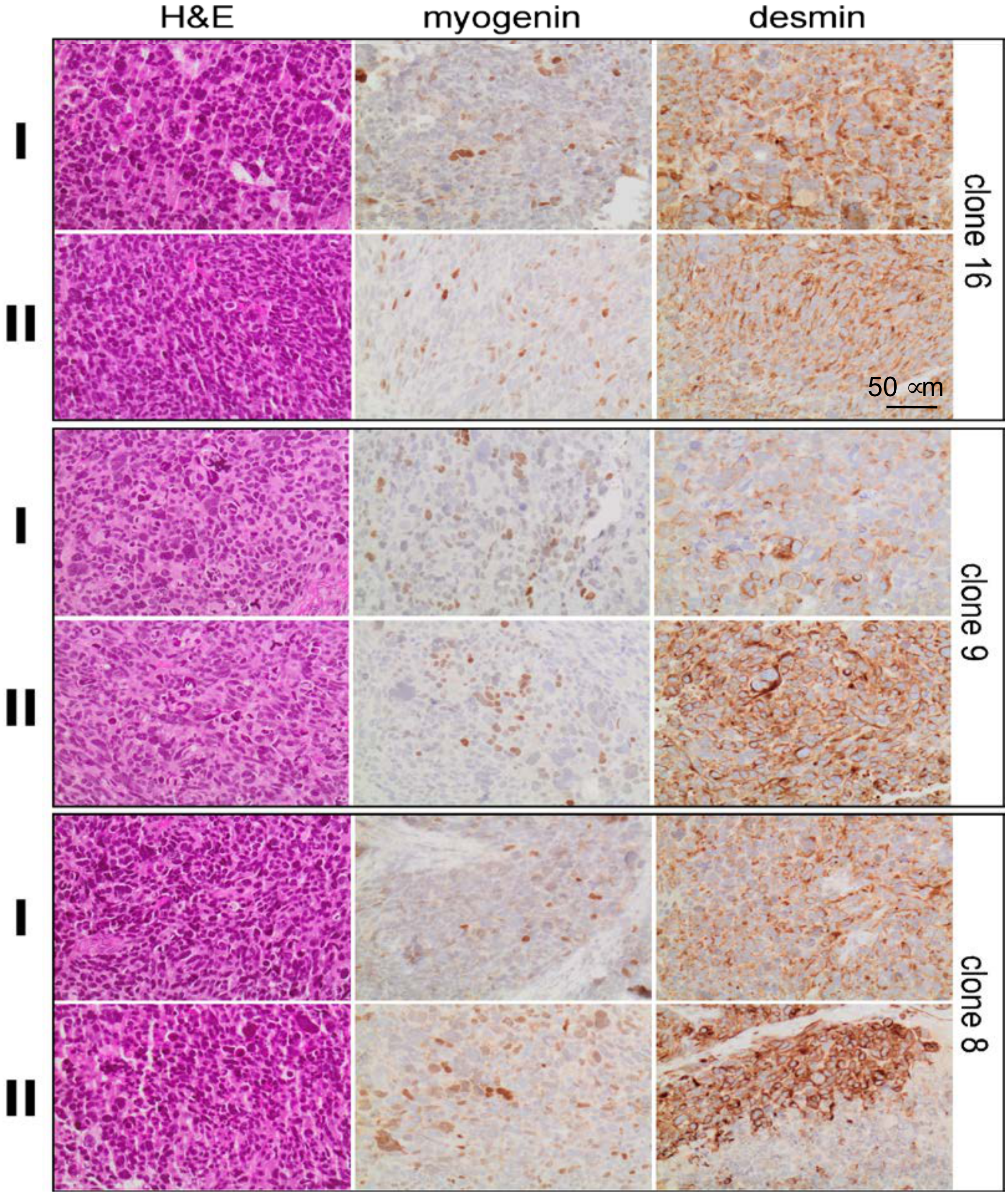

Supplement: Supplementary file 1 [file genes-12-01373-s001.zip › genes-1318311-supplementary.pdf]
